# Exercise and Nutrition Therapy in a Patient With Glottic Cancer Cachexia Undergoing Chemoradiotherapy: A Case Report

**DOI:** 10.7759/cureus.84455

**Published:** 2025-05-20

**Authors:** Kengo Shirado, Yuya Uchiyae, Honoka Kobayashi, Ayane Miyake, Shota Okuno, Masaya Tanaka, Mioko Fukahori, Toshihiro Yamashita

**Affiliations:** 1 Department of Rehabilitation, Iizuka Hospital, Iizuka, JPN; 2 Department of Otorhinolaryngology, Iizuka Hospital, Iizuka, JPN; 3 Department of Internal Medicine, Genki Clinic, Fukuoka, JPN

**Keywords:** cancer cachexia, case report, glottic cancer, medical nutrition therapy, preoperative exercise therapy

## Abstract

Cancer cachexia is a common complication in patients undergoing chemoradiotherapy (CRT) for head and neck cancer (HNC), leading to skeletal muscle loss, decreased physical function, and poor prognosis. Cachexia is frequently observed in patients with HNC, yet evidence supporting effective therapeutic interventions remains limited. We present the case of a man in his 60s with glottic cancer who developed sarcopenia and cancer cachexia following hospitalization for CRT. The patient received a multidisciplinary intervention combining individualized exercise therapy and oral nutritional supplementation tailored to his clinical status. The rehabilitation program was performed five times per week at moderate intensity and included both resistance and aerobic exercises. Following the intervention, the patient maintained skeletal muscle mass and improved physical function despite adverse effects associated with CRT. This case highlights the potential role of multidisciplinary rehabilitation in attenuating functional decline and preventing further muscle loss in patients with HNC undergoing CRT.

## Introduction

Chemoradiotherapy (CRT) is the most-evidenced therapy for locally advanced HNC, including oropharyngeal cancer [1]. However, CRT is associated with weight loss due to treatment-related adverse events [2]. Recent reports indicate that approximately 62% of patients with HNC lose 5% or more of their body weight during CRT treatment [2] and that 71% of patients with HNC with weight loss have decreased skeletal muscle mass [3]. This decrease in skeletal muscle mass and muscle function is defined as sarcopenia [4], which is reported to be present in an average of 39.4% of patients with HNC pre-treatment [5]. Weight loss and nutritional disturbances, common in cancer patients, are collectively referred to as cancer cachexia, a multifactorial syndrome characterized by progressive, severe skeletal muscle loss and a negative protein-energy balance [6]. The prevalence of cancer cachexia in patients with HNC is 36% [7]. Weight loss and malnutrition during CRT in patients with HNC are associated with an increased risk of CRT toxicity and infection, leading to prolonged treatment duration, poor clinical outcomes, increased morbidity and mortality, and decreased QOL [2,8]. Therefore, preventing or ameliorating cachexia in patients with HNC treated with CRT is crucial for improving clinical outcomes and prognosis.

There are numerous reports on the effects of nutrition therapy in HNC. A recent systematic review found that the combination of nutritional counseling and ONS during CRT for patients with HNC was more effective than nutritional counseling alone in improving mortality, treatment tolerance, and QOL. However, currently, only a few reports examine the effects on physical function [9]. Similarly, several reports investigate only the effects of exercise therapy on patients with HNC during CRT. Exercise therapy during CRT effectively improves exercise tolerance and QOL [10]. Recently, the importance of combined rehabilitation and nutrition therapy has been reported [11]. A systematic review of multidisciplinary rehabilitation and nutrition therapy interventions for patients with generalized cancer cachexia found improvement in exercise tolerance and grip strength [12]. However, few studies have focused on patients with HNC. One pilot study suggested that the combination of ONS and resistance training may help suppress muscle loss during CRT [13]. While many existing studies have implemented therapeutic interventions, their outcomes have generally demonstrated only maintenance of body weight and physical function, with few reports showing actual reversal or alleviation of cancer cachexia. Here, we report a case of glottic cancer with cancer cachexia during CRT in which early multidisciplinary intervention involving exercise and nutrition therapy contributed to the preservation of physical function and muscle mass, along with improvement in cachexia.

## Case presentation

Clinical findings

The patient is a male in his late 60s. Before admission, the patient's activities of daily living (ADL) were independent. He had a past medical history of hypertension. He was diagnosed with left glottic cancer after a thorough evaluation and was admitted to the inpatient ward of our hospital for CRT and supportive care. At the time of cancer diagnosis, the patient's Eastern Cooperative Oncology Group performance status grade of 0 indicates that the patient was fully active and could carry out all pre-disease activities without restriction. The cancer was categorized as stage T2N0M0, indicating a tumor of moderate size (T2), no regional lymph node involvement (N0), and no distant metastasis (M0). The classification of T2 was based on supraglottic extension involving both the glottis and supraglottis regions (i.e., two anatomical subsites). Interventions were performed following the 1964 Declaration of Helsinki ethical standards and later amendments. The patient provided informed consent for the publication of this case report.

Diagnostic assessment

The patient's body mass index (BMI) on admission was 22.1 kg/m², and no weight loss was noted prior to hospitalization. However, by the seventh day of CRT (the eighth day of admission), he had lost 3.5% of his body weight. Based on an evaluation of appendicular skeletal muscle mass (ASM), grip strength, and gait speed, the patient was diagnosed with sarcopenia according to the Asia Working Group for Sarcopenia (AWGS) 2019 criteria [4]. In addition, the patient was diagnosed with cancer cachexia based on the AWGC criteria [14], considering weight loss and anorexia assessed by the Simplified Nutritional Appetite Questionnaire (SNAQ), and malnutrition according to the Global Leadership Initiative on Malnutrition (GLIM) criteria [15]. Bioelectrical impedance analysis (Inbody 770, Inbody Co., Seoul, Korea) was used to evaluate the patient's ASM and body water balance (extracellular water/total body water; ECW/TBW) at the start of exercise therapy. Grip strength was measured using a Smedley hand dynamometer (TTM, Tokyo, Japan) with the patient standing and the upper limb hanging down along the trunk, twice on each side, and the maximum value was taken [4]. The gait speed was measured by instructing the patient to walk a distance of 4 m, and the starting point was one meter in front of the participant [16].

Therapeutic interventions

**Figure 1:**
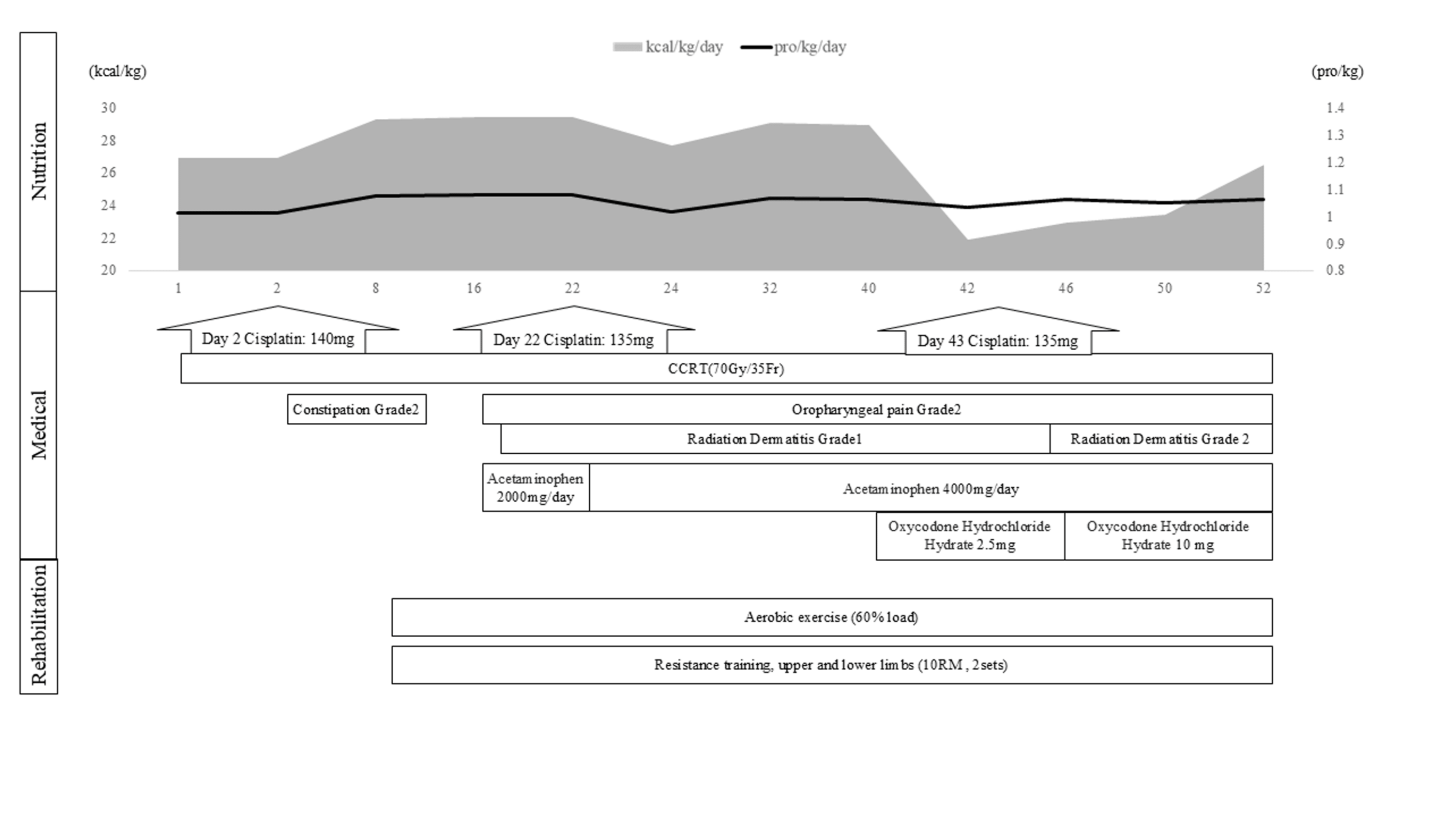
Timeline of the patient’s treatment. CDDP, cis-diamminedichloroplatinum; CCRT, concomitant chemoradiotherapy Concurrent chemoradiotherapy was initiated on day two of admission. Despite experiencing adverse events such as oropharyngeal pain and radiation dermatitis, the patient underwent multidisciplinary intervention involving exercise and nutrition therapy alongside CRT and completed the planned treatment without interruption.

The treatment course is shown in Figure 1. The patient started CRT treatment the day after admission. Chemotherapy was administered with cisplatin on days 2, 22, and 43. Radiotherapy was administered with a total dose of 70 Gy (35 Fr). Adverse events from CRT included constipation (grade 2), oropharyngeal pain (grade 2), and radiation dermatitis (grades 1-2). Acetaminophen and opioids were used as needed to control sore throat.

On the eighth day of admission, the patient started exercise therapy with a physiotherapist. The main goal was to improve sarcopenia and cachexia. Exercise therapy primarily consisted of aerobic and resistance training targeting the upper and lower limb muscles, performed for 40-60 minutes per day, five to six days per week during hospitalization. Aerobic exercise was conducted for 10-15 minutes using an ergometer bicycle at a target heart rate corresponding to 60% of the heart rate reserve, calculated using the Karvonen formula. Resistance training was performed using weight-training machines and focused on the major muscle groups of the upper and lower extremities, including leg press, leg extension, and chest press. The training load was adjusted to approximately the patient’s 10-repetition maximum, and each exercise was performed for two sets. Subjective muscle strength during exercise was assessed using a modified Borg scale. In order to ensure continued effective exercise, the exercise therapy program was adjusted as needed to ensure that overall body and muscle fatigue during and after exercise were less than a modified Borg scale of 4 (low intensity).

At admission, the patient's goal was body weight maintenance, with a daily energy intake of 26.9 kcal/ideal body weight (IBW)/day and protein intake of 1.01 g/kg IBW/day, based on institutional protocol aligned with the European Society for Clinical Nutrition and Metabolism (ESPEN) guidelines [17]. On the eighth day after beginning exercise therapy, a weight loss of 3.5% was observed, so the nutritional care plan was revised in consultation with the dietitian and primary physician, with the goal to gain approximately 1 kg per month and maintain ASM. The registered dietitian and the patient's primary care physician shared information on the patient's goals, treatment measures, and monitoring results, and the registered dietitian revised the plan as needed (at least once a week). The registered dietitian monitored daily body weight, body composition, and blood chemistry test results and adjusted the diet so that the daily energy intake was approximately 30 kcal/kg IBW/day and protein intake was 1.1 g/kg IBW/day. In addition to routine assessments by the dietitian, blood tests were conducted at regular intervals based on the clinical judgment of the attending physician. The results were used to monitor the patient's nutritional and inflammatory status and to guide adjustments to the care plan. Furthermore, two oral nutritional supplements (ONS) were administered to promote muscle protein synthesis; Meibalance Mini: 125 mL, 200 kcal, protein: 7.5 g, branched-chain amino acids: 1.92 g, vitamin D: 20 µg (Meiji Co., Ltd. Tokyo, Japan), and Reha-Time Jelly: 120 mL, 100 kcal, protein: 10 g, branched-chain amino acids: 2.5 g, vitamin D: 20 µg (Morinaga Milk Industry Co Ltd., Tokyo, Japan) within 30 minutes of the end of rehabilitation, and one of each taken at a time chosen by the patient. These ONS are commercially available and are frequently used in clinical practice.

Follow-up and outcomes

After starting treatment, the Eating Assessment Tool (EAT-10) and the EuroQol 5 Dimensions 5-Level (EQ-5D-5L) scores gradually worsened due to an adverse event, oropharyngeal pain (CTCAE ver5.0, grade 2). However, the patient could complete treatment without discontinuing physical therapy, fasting, or enteral nutrition. Compared to the start of rehabilitation (day eight), BMI improved from 21.3 kg/m^2^ to 21.6 kg/m^2^ on day 40, but from day 40 onward, food intake declined, dropping BMI to 21.1 kg/m^2^ on the day before discharge (day 52). Nevertheless, the rate of weight loss since the beginning of rehabilitation was only 0.82%. Compared to the start of rehabilitation, on the day before discharge (day 52), ASM was maintained. Grip strength decreased slightly, by 2.69% on the right side (from 33.4 kg to 32.5 kg) and 3.29% on the left side (from 33.4 kg to 32.3 kg). In contrast, gait speed improved by 17.7% (from 0.96 m/s to 1.13 m/s), and the five-repetition sit-to-stand test (5STS) improved by 7.2% (from 9.65 seconds to 8.96 seconds). Throughout hospitalization, the patient’s extracellular water to total body water (ECW/TBW) ratio remained within the normal range (0.36-0.39). Meanwhile, the phase angle (PA), an indicator of cellular health and membrane integrity, showed a gradual decline by -13.7% (from 5.1° to 4.4°) during treatment. The patient’s sarcopenia and cancer cachexia were alleviated (Tables 1-2).

**Table 1:** Timeline of the patient’s physical and nutritional statuses. ASM, appendicular skeletal muscle mass; BMI, body mass index; ECW/TBW, extracellular water/total body water; PA, phase angle; 5STS, five-repetition sit-to-stand test; FIM, functional independence measure; SNAQ, Simplified Nutritional Appetite Questionnaire; EQ-5D-5L, EuroQol 5 dimensions 5-level; CRP, C-reactive protein

Number of days of hospitalization	1	8	16	32	40	52	Variation Rate	Reference Range
							(day 8-52)	
Body weight (kg)	63.1	60.9	60.6	61.3	61.6	60.4	-0.82%	N/A
BMI (kg/m^2^)	22.1	21.3	21.2	21.5	21.6	21.1	-0.82%	18.5-22.9 (Asian)
ASM (kg/m^2^)	-	6.5	6.6	6.4	6.4	6.5	0.00%	>7.0 (men), >5.7 (women)
Body fat (kg)	-	15.5	14.4	15.6	15.7	14.5	-6.45%	N/A
ECW/TBW	-	0.384	0.381	0.384	0.385	0.389	1.30%	0.36-0.39
PA (°)	-	5.1	4.9	4.7	4.7	4.4	-13.73%	N/A
Grip strength, Rt (kg)	-	33.4	-	-	-	32.5	-2.69%	>28 (men), >18 (women)
Grip strength, Lt (kg)	-	33.4	-	-	-	32.3	-3.29%	
Gait speed (m/sec)	-	0.96	-	-	-	1.13	18.04%	>1m/s
5STS	-	9.65	-	-	12.75	8.96	-7.15%	<12 sec
FIM, motor items (points)	-	85	-	-	-	85	-	N/A
FIM, cognitive items (points)	-	33	-	-	-	33	-	N/A
SNAQ	-	14	-	-	-	-	-	>13.5
EQ-5D-5L	-	1	0.955	0.955	0.869	0.869	-	N/A
Hemoglobin (g/dL)	-	15	13.1	12.6	12.6	11.5	-	N/A
CRP (mg/dL)	-	<0.02	0.29	0.86	0.14	0.91	-	<0.3
Albumin (g/dL)	-	3.8	3.7	3.7	4.2	3.9	-	N/A

**Table 2:** Detail of the patient’s EAT-10. EAT-10, eating assessment tool

Number of days of hospitalization	8	16	24	32	40	52
							My swallowing problem has caused me to lose weight.	0	0	0	0	0	1
My swallowing problem interferes with my ability to go out for meals.	0	0	0	0	1	1
Swallowing liquids takes extra effort.	0	0	0	1	2	1
Swallowing solids takes extra effort.	0	0	0	1	2	1
Swallowing pills takes extra effort.	0	0	0	1	2	1
Swallowing is painful.	0	0	1	1	3	1
The pleasure of eating is affected by my swallowing.	0	1	1	1	2	2
When I swallow food sticks in my throat.	0	0	0	1	2	1
I cough when I eat.	0	1	1	1	1	0
Swallowing is stressful.	0	0	0	1	1	1
Total	0	2	3	8	16	10

## Discussion

Two critical clinical implications can be suggested in this paper. First, the combination of exercise therapy and nutrition therapy may improve physical function in patients with glottic carcinoma cachexia on CRT. Second, the combination of exercise therapy and nutrition therapy may not only improve physical function, but also cancer cachexia may be improved in patients with glottic carcinoma cachexia on CRT.

Exercise therapy combined with nutrition therapy improved physical function in patients with glottic carcinoma cachexia on CRT. Exercise therapy for HNC patients on CRT effectively improves six-minute walk distance and QOL [10], but there are no reports of improvement in muscle strength or muscle mass. These results may be partly due to the fact that most of the previous studies did not provide sufficient exercise load, as most transitioned to home exercise after the eighth week of intervention. Regarding the efficacy of nutrition therapy during CRT in patients with HNC, a recent systematic review reported that nutritional counseling combined with ONS improved mortality, treatment tolerance, and QOL but had little effect on improving muscle strength [9]. However, this previous study did not examine the specifics of counseling and ONS. In summary, the effects of exercise and nutrition therapy alone in patients with HNC are limited, and evidence for effects on physical function is lacking. On the other hand, the importance of combined rehabilitation and nutrition therapy has been reported in recent years [11,18]. A systematic review of multidisciplinary interventions with rehabilitation and nutrition therapy for patients with common cancer cachexia acknowledges some evidence of improvement in physical function, such as grip strength, but the details of nutritional and exercise interventions are not clear [12]. In addition, studies on head and neck cancer patients are scarce, with only one pilot study suggesting a trend toward alleviation of muscle mass loss [13]. In our present case, the patient's physical function, including gait speed, improved despite ongoing CRT. Although a slight decline in grip strength was observed (-2.69% in the right hand, -3.29% in the left hand), it did not exceed the minimally clinically important difference for grip strength (approximately 5-6.5 kg) reported in a systematic review of cancer prehabilitation trials. Thus, the changes in grip strength were within a clinically acceptable range, indicating stabilization of muscle strength [19]. We have seen one case that improved physical function (grip strength and walking speed) in a lung cancer patient undergoing treatment for cancer cachexia when exercise therapy was combined with nutrition therapy [20]. In this case, the patient's physical function was improved by multidisciplinary nutritional management, including ONS and aggressive exercise therapy.

A combination of aggressive exercise and nutrition therapy may improve cancer cachexia in patients with glottic carcinoma on CRT. Cancer cachexia is primarily driven by tumor-induced inflammatory and metabolic changes, along with secondary factors, such as treatment-related nausea, taste alterations, reduced physical activity, and psychological stress [21]. In particular, inflammatory cytokines, including tumor necrosis factor-α and interleukin-6, promote skeletal muscle catabolism. In this case, the patient developed marked weight loss and cancer cachexia within eight days of admission, likely due to these factors combined with insufficient nutritional intake. The prevalence of cachexia increases significantly by the end of CRT in patients with HNC [22], and weight loss during treatment is associated with worse clinical outcomes [2,8]. Early nutritional intervention is recommended to prevent severe malnutrition [17], and multidisciplinary rehabilitation incorporating both exercise and nutritional support has been advocated. However, as noted in previous systematic reviews, evidence supporting improvements in nutritional status through combined interventions in advanced cancer remains limited (grade D) [12]. In a prior pilot study utilizing a similar exercise and nutrition protocol, patients undergoing CRT experienced approximately 7% weight loss and 5% skeletal muscle mass loss over 14 weeks [13]. In contrast, our patient lost only 0.82% of body weight and maintained ASM during CRT. This degree of weight loss remained well below the 2% threshold for clinically significant cachexia progression proposed by the AWGC [14]. Although direct comparisons are limited due to differences in reported energy and protein intakes, our findings suggest that individualized adjustment of nutritional (30 kcal/kg IBW/day, 1.1 g/kg IBW/day) and exercise regimens may contribute not only to the preservation of physical function and body composition but also to the alleviation of cancer cachexia. The patient's phase angle (PA) progressively decreased during hospitalization. Although previous studies have suggested that PA reflects cellular integrity and nutritional status, the magnitude of change considered clinically significant remains unclear [23]. In this case, combining individualized exercise and nutrition therapy contributed to minimizing weight loss and maintaining ASM despite the inflammatory burden. In the present case, the patient maintained ASM and ECW/TBW and improved physical function despite a decrease in PA, suggesting that multidisciplinary intervention may have been effective in HNC patients undergoing CRT. In addition, during CRT, the patient's C-reactive protein (CRP) levels consistently exceeded 0.5 mg/dL, meeting the inflammatory criterion for cancer cachexia defined by the AWGC. Although CRP levels fluctuated between 0.02 and 0.91 mg/dL, suggesting a sustained inflammatory state, significant body weight loss and skeletal muscle mass loss were not observed. Recent studies also suggest a significant association between high CRP and sarcopenia in cancer patients [24]. This case provides novel insight into the potential benefits of early, individualized multidisciplinary intervention during CRT in patients with HNC. As this is a single-case report, causality cannot be definitively established. The observed improvements may reflect the effects of the combined intervention, individual patient characteristics, or both. Further research is needed to validate the generalizability of these findings.

## Conclusions

This case suggests that a combination of exercise and nutrition therapy may help attenuate functional decline and prevent further skeletal muscle loss in patients with glottic cancer undergoing CRT. Further research is needed to clarify the effectiveness of this multidisciplinary approach in managing cancer cachexia in patients with HNC.
